# Sexual experiences of married HIV positive women in Osogbo, southwest Nigeria: role of inappropriate status disclosure

**DOI:** 10.1186/s12905-015-0164-7

**Published:** 2015-02-07

**Authors:** Daniel Adebode Adekanle, Samuel Anu Olowookere, Ayobami David Adewole, Najemdeen Ajao Adeleke, Emmanuel Akintunde Abioye-Kuteyi, Macellina Yinyinade Ijadunola

**Affiliations:** Department of Obstetrics and Gynaecology, Ladoke Akintola University Teaching Hospital, Osogbo, Nigeria; Department of Community Health, College of Health Sciences, Obafemi Awolowo University, Ile-Ife, Nigeria; Department of Community Medicine, Bowen University Teaching Hospital, Ogbomoso, Nigeria; Department of Obstetrics and Gynaecology, Osun State University, Osogbo, Nigeria

**Keywords:** Health care workers, Nulliparity, Marital sex deprivation, HIV positive women, Nigeria

## Abstract

**Background:**

Worldwide heterosexual sex is the most common mode of HIV transmission, with the marital heterosexual route becoming a major contributor in sub-Sahara Africa. This study examined the role of inappropriate HIV status disclosure, after diagnosis, on marital sexual experiences of HIV positive women.

**Methods:**

The study employed a descriptive cross-sectional design. An interviewer administered questionnaire that elicited information about HIV status disclosure to partners, sexual experiences, condom use and parity was administered to 122 married women living with HIV/AIDS. Participants were referred from peripheral health centres to receive comprehensive HIV care at the State Specialist Hospital, Osogbo, Nigeria.

**Results:**

Mean age (SD) of respondents was 33.8 (8.9) years. Only 23.8% of partners had HIV screening, with 3.3% being HIV positive. A majority (62%) of respondents reported experiencing marital sex deprivation since their partners became aware of their HIV status. There was a reported rejection (74.3%) of condom use by partners during sexual intercourse. Fear of becoming infected (85.7%) and blaming the women for their positive status (85.7%) were the main reasons the respondents gave for being sexually deprived by their partners.

**Conclusion:**

Inappropriate status disclosure due to poor HIV counseling and testing (HCT) practices resulted in sexual deprivation of married HIV positive women. Adequate training and retraining of health care workers on HCT and HIV status disclosure will reduce experience of sexual deprivation among married HIV positive women.

## Background

The sub-Sahara African region is home to most people living with Human Immunodeficiency Virus/Acquired Immunodeficiency Syndrome (PLWHA), with Nigeria having the third highest population worldwide, after South Africa and India [1,2]. Since 1986, when the first Human Immunodeficiency Virus/Acquired Immunodeficiency Syndrome (HIV/AIDS) case was reported in Nigeria, the disease has spread to every Nigerian community [3-5]. Although HIV/AIDS affects all age groups in Nigeria, it occurs mostly among the sexually active population aged 20 to 29 years [4,6,7].

The most common mode of transmission of HIV/AIDS in Nigeria is unprotected heterosexual intercourse [5-8]. Marital sex is becoming a major route of HIV transmission due to the high sexual activity among couples who desire to have children but have never had HIV counseling and testing [9,10] and poor condom use among couples [11,12]. Other modes of transmission include blood and blood products, injecting drug use and mother to child transmission during pregnancy, labour and breast feeding [2,4,8].

Worldwide, most HIV positive women experience stigma and discrimination from their family members. Studies have shown that HIV positive women are frequently referred to as ‘vectors’, ‘diseased’ and ‘prostitutes’ while most HIV positive men are spared this highly stigmatising treatment [13,14]. HIV/AIDS stigma commonly occurs as the disease is perceived as being contracted only through extramarital sex. Hence, HIV positive patients experience stigma from close family members, colleagues in places of work, schools and religious gatherings where many still recognize the virus as “a curse” and HIV positive people as “sinners” [15-18].

HIV counseling and testing (HCT) is the gateway to identifying HIV positive clients that require comprehensive care, including antiretroviral treatment, thereby reducing the probability of spreading the infection to their contacts [19]. Currently, HCT is being carried out in most health centres in the 30 Local Government Areas of Osun State with HIV positive cases being referred to the Osun State Specialist hospital, Osogbo to access antiretroviral treatment.

Sexual intercourse is important in a married relationship; hence, denying a partner sex is discouraged while unprotected sexual intercourse is perceived in many cultures as strengthening the marriage institution [9]. Also, condom use is sometimes discouraged in marital sex because of couple’s desire for children and its’ use is equated with unfaithfulness and unnecessary indulgence by partners. Many marriages break down due to unmet sexual needs of couples [18,20-22].

This study examined the role of inappropriate HIV status disclosure on the marital sexual experiences of HIV positive women accessing care at the Osun State Specialist Hospital, Osogbo, Nigeria. The results will provide empirical evidence that may prove useful in redesigning HCT programmes in ways that will reduce experiences of stigma and discrimination among married HIV positive women.

## Methods

The Osun State Government, in collaboration with the Global HIV/AIDS Initiative Nigeria, established the antiretroviral (ART) centre at the State Specialist Hospital, Osogbo to provide comprehensive HIV/AIDS care for HIV positive patients, and receive referral of HIV positive clients from all health care facilities in Osun and other neighbouring states. The centre opens daily from 8:00 a.m. to 4:00 p.m. while emergency services are provided on 24 hour basis. It provides highly active antiretroviral therapy (HAART) to eligible clients living with HIV/AIDS every Monday and Wednesday. The centre is headed by the ART coordinator with the support of other health care workers [23].

Ethical approval for the study was obtained from the Osun State Specialist Hospital Ethics and Research Committee. A written informed consent was obtained from each respondent before administering the questionnaire. Confidentiality of collected data was maintained throughout the study as only the investigators stored data generated from the research.

This study employed a descriptive cross-sectional design. The study population included all 122 eligible currently married adult HIV positive women receiving care at the clinic from May to August 2011. Clients who were too ill to be interviewed and non-consenting clients were excluded from the study.

A semi-structured, pre-tested, interviewer-administered questionnaire was applied to the respondents. It elicited information on socio-demographic characteristics, sources of HIV status disclosure and whether consent was obtained from the respondents before disclosure; sexual experiences since status disclosure, parity and condom use during marital sex. Inappropriate HIV status disclosure was defined as disclosure of client’s HIV status to the partner without client’s consent after undergoing HCT, while sexual deprivation was defined as refusal of sex by the partner whenever the respondent wanted to have sex. The interview was conducted individually in a private room designated for the research.

The data were entered and analyzed using SPSS version 16. Simple descriptive and inferential statistics were performed and the results presented in tables. A binary logistic regression model was used to examine the relationship between experience of marital sex deprivation (main outcome variable) and socio-demographic variables, parity and partners’ awareness of respondents’ HIV status (explanatory variables). Results were expressed as odds ratios (ORs) and 95% confidence intervals (CIs). Tests were considered significant for a p-value of <0.05.

## Results

Mean age (SD) of respondents was 33.8 (8.9) with a range of 21 - 49 years. Most respondents were Yoruba (91.8%), half were traders and 42.6% had secondary education. All the respondents were heterosexuals, while 27 (22.1%) of them were nullipara (Table 1).

**Table 1:** **Sociodemographic characteristics of respondents**

**Socio-demographic distribution**	**Frequency (N = 122)**	**%**
**Age group (years)**		
20 – 29	49	40.2
30 –39	32	26.2
40 and above	41	33.6
**Highest level of education**		
None	4	3.3
Primary	20	16.4
Secondary	52	42.6
Tertiary	46	37.7
**Parity**		
0	27	22.1
1 – 4	76	62.3
5 and above	19	15.6
**Occupation**		
Trader	61	50.0
Artisan	18	14.8
Civil servant	10	8.2
Farming	10	8.2
Unemployed	23	18.9
**Ethnicity**		
Yoruba	112	91.8
Others	10	8.2
**Sexual orientation**		
Heterosexual	122	100

Table 2 shows that 29 (23.8%) respondents’ partners had had HIV screening done with 4 (3.3%) being HIV positive. All HIV positive partners and 92% of HIV negative partners were aware of respondents’ HIV status.

**Table 2:** **Association between partners’ awareness of respondent HIV status and partners’ HIV status**

**Variable**	**Partners’ awareness of respondent HIV status**		**Total**	*p-value
	**Yes (%)**	**No (%)**		
**Partners' HIV status**				<0.001
Positive	4 (100)	0 (0)	4 (100)	
Negative	23 (92)	2 (8)	25 (100)	
Don’t know	1 (1)	92 (99)	93 (100)	
Total	28 (23.0)	94 (77.0)	122 (100)	

*Fisher’s exact test.

Table 3 shows that 76 (62.3%) respondents reported experiencing marital sex deprivation since their HIV positive status were disclosed to their partners. Eighty seven respondents (71.3%) did not offer to use a condom before sex, however there was a high rejection rate (74.3%) of condom use among respondents’ partners when they did offer to use one. The factors that were significantly associated with experience of marital sex deprivation were younger age (87.8% vs. 78.1% vs. 19.5%; p < 0.001), high education (75.5% vs. 8.3%; p < 0.001), partners’ awareness of respondents’ HIV status (100% vs. 51%; p < 0.001) and nulliparity (100% vs. 51.3% vs. 52.6%; p < 0.001).

**Table 3:** **Association between respondents’ sociodemographic variables, awareness of HIV status, parity and marital sexual deprivation**

**Variable**	**Sexually deprived No (%)**	**Not sexually deprived No (%)**	***χ*** ^**2**^	**df**	**p-value**
	**(n**_**1**_ **= 76)**	**(n**_**2**_ **= 46)**			
**Age group**					
20 – 29	43 (87.8)	6 (12.2)	48.887	2	<0.001
30 – 39	25 (78.1)	7 (21.9)			
40 and above	8 (19.5)	33 (80.5)			
**Level of education**					
None/primary	2 (8.3)	22 (91.7)			<0.001*
Secondary/tertiary	74 (75.5)	24 (24.5)			
**Partners’ awareness of respondents’ HIV status**					
Yes	28 (100)	0 (0.0)			<0.001*
No	48 (51.1)	46 (48.9)			
**Parity**					
0	27 (100)	0 (0.0)			<0.001*
1 – 4	39 (51.3)	37 (48.7)			
5 and above	10 (52.6)	9 (47.4)			
**Partner’s attitude to condom use during marital sex**					
Accept condom	6 (66.7)	3 (33.3)			0.883*
Refuse condom	17 (65.4)	9 (34.6)			
Condom not offered	53 (61)	34 (39)			

*Fisher’s exact test.

Table 4 shows that only 28 (23%) respondents reported that their partner knew their HIV status. These women reported that health care workers had disclosed their HIV status to their partners or family members without obtaining their consent. Few women (14.3%) reported that their partners’ awareness of their HIV status helped strengthen their marital relationships. Furthermore, the fear of their partners contracting HIV infection (85.7%) and their partners blaming them for testing HIV positive (85.7%) were the main reasons the respondents gave for being sexually deprived by their partners. Other reasons given for their sexual experience included respondents’ illness (75%), pregnancy (50%) and their partners having other sexual partners (28.6%).

**Table 4:** **Selected variables of HIV status disclosure to partners**

**Variable**	**Frequency**	**%**
	**n = 28**	
**Source of HIV status disclosure to partner**		
Health care workers	27	96.4
Family	1	3.6
**Consent sought before disclosing status to partner**		
Yes	3	10.7
No	25	89.3
**Partner’s awareness of respondent’s HIV status had positive effect on marriage**		
Yes	4	14.3
No	24	85.7
*Reasons given for sexual behavior of partner		
Partners’ fear of contracting HIV infection	24	85.7
Partners blame respondents for contracting HIV infection	24	85.7
Partner’s illness	21	75.0
Respondent pregnant	14	50.0
Has other wife/wives/partner	8	28.6
Don’t know	4	14.3

*Multiple responses.

The binary logistic regression analysis of selected variables predicting marital sexual deprivation among HIV positive women is displayed in Table 5. Significant predictors of marital sexual deprivation among respondents included respondents’ age <40 years (OR = 1.923, 95% CI = 1.441-2.618, p < 0.0001), higher education (OR = 34.483, 95% CI = 2.857-166.667, p < 0.0001) and not having had any children (OR = 1.862, 95% CI = 1.449-2.392, p < 0.0001).

**Table 5:** **Binary logistic regression analysis of select variables predicting marital sex deprivation among HIV positive women**

**Variable**	**Odds ratio; 95% CI; p-value**
**Respondent’s age (years)**	
21-39	1.923; 1.441-2.618; <0.0001
≥40 (ref)	1
**Level of education**	
None/primary (ref)	1
Secondary/tertiary	34.483; 2.857-166.667; <0.0001
**Parity**	
None	1.862; 1.449-2.392; <0.0001
≥1 (ref)	1
**Partners’ awareness of respondents’ HIV status**	
Yes	1.55; 0.892-10.564; 0.998
No (ref)	1

Ref = reference.

## Discussion

This study documented the role of inappropriate HIV status disclosure on marital sexual experiences, parity and condom use in marital sex among couples with HIV positive wives. It reported that most HIV positive women’s statuses were disclosed to their partners without the woman’s consent and that they experienced sexual deprivation once their HIV statuses were known by their partners. This sexual deprivation could result from the partners’ awareness that sexual transmission is the most common mode of transmission of the virus. A recent study among these HIV positive women reported high levels of intention to have children [23] and this could explain the reason for their concern about their partners’ behaviour since becoming aware of their HIV status.

This study also reported that most women interviewed did not offer to use a condom during sex with their partner and most partners offered the condom refused to use it. Previous studies have similarly shown low condom use among the HIV positive population [12,24-26]. This lack of use of condoms could result from undesirability of the condom in marriage and perceived decrease in sexual pleasure from condom use [11,12,15]. Also, the disposition of the male partners could lead to their seeking sexual intercourse from other women they perceived to be uninfected. Marital sex deprivation was reportedly more common among respondents whose partners refused to use a condom. The influence of religious belief on condom refusal cannot also be overlooked [26].

The study findings indicated that some HIV positive women reported experiencing sexual deprivation from their partners because of pregnancy, illness and the partner having other sexual partners. However, no respondent who indicated being sexually deprived by their partner willingly abstained from marital sex. Various studies have shown that inappropriate HIV status disclosure could result in the HIV positive individual experiencing stigma and discrimination [17,20]. Sexual deprivation from husbands experienced by married HIV positive women as reported in this study could result in marital disharmony. Therefore, it is suggested that health workers should always seek and obtain consent from HIV positive clients before disclosing their HIV status to their partners and family members. Further, health workers should receive appropriate training and re-training on HCT and HIV disclosure, especially to partners and family members of HIV positive women. Stigma and discrimination will be reduced with proper public education and couple counselling, thereby eliminating such untoward consequences [20]. Although HIV disclosure is encouraged, it must be done after proper counselling and the consent of HIV positive women as well as their partners has been obtained in order to prevent stigma and discrimination.

This study showed that uptake of HCT was low among partners of women living with HIV/AIDS in this environment; hence, some partners’ HIV status remained unknown. This might not be unconnected with the manner the partners became aware of their wives’ HIV status. In this current situation, partners who are already infected may not know their status, and therefore might not access treatment, care and support resulting in continued spread of the virus to other sexual contacts. Also, partners who are already infected without knowing may progress to full blown AIDS. Moreover, HIV negative partners could become infected if they have not been counselled on HIV prevention. In order to curb further spread of the virus, it is necessary to trace these partners and refer them for HCT services.

Sexual deprivation was reportedly more common among respondents less than 40 years, those with higher education and nulliparous women. This finding could be because the younger age group is usually more sexually active and further explains the high HIV prevalence among them. Also women with lower education are more likely to be culturally inclined not to report their sexual experiences so as not to be seen as being promiscuous, while women with higher education could be more assertive.

At the time of this write up, there was a dearth of literature on the role of inappropriate HIV status disclosure on marital sexual experiences, parity and condom use in marital sex among couples with HIV positive wives. Also, our study draws attention to the need for appropriate HIV status disclosure and the sexual health needs of HIV positive women especially among nulliparous women and the implications for the society. However, this study was not without some limitations. The authors upheld every ethical standard (assuring respondents of the confidentiality of information provided) in obtaining accurate information from respondents in the HIV treatment centre, and for these reasons could not interview their partners. Furthermore, the sensitive nature of sex and HIV infection, especially in the study environment, could lead some respondents to withhold more useful information about their sexual experiences or simply give socially desirable responses.

Further investigation of the role of inappropriate HIV status disclosure on marital sexual experiences is required for planning sexual health needs for couples with HIV positive wives. The relationship between higher education and marital sex deprivation found in the study needs further investigation.

## Conclusion

Marital sexual deprivation was common among young nulliparous married women after inappropriate HIV status disclosure to their male partners. Early couple counselling and partner testing may reduce stigma and encourage condom use even among couples desiring children. Adequate training and retraining of health care workers on HCT, HIV status disclosure and couple counseling will reduce experience of marital sex deprivation among couples with HIV positive wives.
